# The Development of a Consistent Europe-Wide Approach to Investigating the Economic Impact of Myalgic Encephalomyelitis (ME/CFS): A Report from the European Network on ME/CFS (EUROMENE)

**DOI:** 10.3390/healthcare8020088

**Published:** 2020-04-07

**Authors:** Derek F.H. Pheby, Diana Araja, Uldis Berkis, Elenka Brenna, John Cullinan, Jean-Dominique de Korwin, Lara Gitto, Dyfrig A Hughes, Rachael M Hunter, Dominic Trepel, Xia Wang-Steverding

**Affiliations:** 1 Society and Health, Buckinghamshire New University, High Wycombe HP11 2JZ, UK; 2 Department of Dosage Form Technology, Faculty of Pharmacy, Riga Stradins University, Dzirciema Street 16, LV-1007 Riga, Latvia; Diana.Araja@rsu.lv; 3 Institute of Microbiology and Virology, Riga Stradins University, Dzirciema Street 16, LV-1007 Riga, Latvia; Uldis.Berkis@rsu.lv; 4 Department of Economics and Finance, Università Cattolica del Sacro Cuore, Largo Agostino Gemelli 1, 20123 Milan, Italy; elenka.brenna@unicatt.it; 5 School of Business & Economics, National University of Ireland Galway, University Road, H91 TK33 Galway, Ireland; john.cullinan@nuigalway.ie; 6 Internal Medicine Department, University of Lorraine, 34, cours Léopold, CS 25233, F-54052 Nancy CEDEX, France; jean-dominique.dekorwin@univ-lorraine.fr; 7 University Hospital of Nancy, Rue du Morvan, 54511 Nancy, France; jd.dekorwin@chru-nancy; 8 Department of Economics, University of Messina, Piazza Pugliatti 1, 98122 Messina, Italy; lara.gitto@unime.it; 9 Centre for Health Economics & Medicines Evaluation, Bangor University, Bangor LL57 2PZ, UK; d.a.hughes@bangor.ac.uk; 10 Institute of Epidemiology & Health, Royal Free Medical School, University College London, London NW3 2PF, UK; r.hunter@ucl.ac.uk; 11 School of Medicine, Trinity College Dublin, College Green, D02 PN40 Dublin 2, Ireland; trepeld@tcd.ie; 12 Global Brain Health Institute, School of Medicine, Trinity College Dublin, College Green, D02 PN40 Dublin 2, Ireland; 13 Warwick Medical School, University of Warwick, Coventry CV4 7AL, UK; xiasteverding@gmail.com

**Keywords:** ME/CFS, economic impact, cost-of-illness studies, economic evaluation, healthcare systems

## Abstract

We have developed a Europe-wide approach to investigating the economic impact of Myalgic Encephalomyelitis/Chronic Fatigue Syndrome (ME/CFS), facilitating acquisition of information on the economic burden of ME/CFS, and international comparisons of economic costs between countries. The economic burden of ME/CFS in Europe appears large, with productivity losses most significant, giving scope for substantial savings through effective prevention and treatment. However, economic studies of ME/CFS, including cost-of-illness analyses and economic evaluations of interventions, are problematic due to different, arbitrary case definitions, and unwillingness of doctors to diagnose it. We therefore lack accurate incidence and prevalence data, with no obvious way to estimate costs incurred by undiagnosed patients. Other problems include, as for other conditions, difficulties estimating direct and indirect costs incurred by healthcare systems, patients and families, and heterogeneous healthcare systems and patterns of economic development across countries. We have made recommendations, including use of the Fukuda (CDC-1994) case definition and Canadian Consensus Criteria (CCC), a pan-European common symptom checklist, and implementation of prevalence-based cost-of-illness studies in different countries using an agreed data list. We recommend using purchasing power parities (PPP) to facilitate international comparisons, and EuroQol-5D as a generic measure of health status and multi-attribute utility instrument to inform future economic evaluations in ME/CFS.

## 1. Introduction

Myalgic Encephalomyelitis/Chronic Fatigue Syndrome (ME/CFS) is a poorly understood, serious, complex, multi-system disorder, characterized by symptoms lasting at least six months, with severe incapacitating fatigue not alleviated by rest, and other symptoms, many autonomic or cognitive in nature, including profound fatigue, cognitive dysfunction, sleep disturbances, muscle pain, and post-exertional malaise, which lead to substantial reductions in functional activity and quality of life [1,2,3]. Symptomatology, severity and disease progression are extremely variable. ME/CFS most commonly occurs between the ages of 20 to 50 years, butan affect all age groups, while some three quarters of patients are female [4,5,6]. There are no Europe-wide prevalence data, but if the commonly held belief that there are some 250,000 sufferers in the UK is correct [7], then there may be some two million patients in Europe as a whole.

In terms of estimating the economic costs of ME/CFS, the current state of the art, and its historical development, have recently been described by Brenna and Gitto, who carried out a comprehensive literature review [8,9]. The literature was reviewed chronologically and detailed the evolution of economic studies of ME/CFS, including cost of illness studies and economic evaluations (e.g., cost effectiveness and cost utility analyses) of specific interventions. The authors also drew attention to the failure of many patients with the condition to be correctly diagnosed, which renders problematic attempts to determine the economic burden of the disease. Another problem they identified was that of determining direct, indirect and intangible costs in a context where there is lack of agreement over, and inconsistent use of, case definitions. This, in turn, is reflected in a lack of agreement regarding the incidence and prevalence of the condition. The prevalence in developed countries appears to be within the range of 0.2–1%, but this is highly dependent on case definition, while there is published research on ME/CFS in only a small number of countries, notably Australia, USA and the UK. Brenna and Gitto [8] concluded that a clearer definition of the population prevalence of ME/CFS would make it easier to reach a general consensus on its economic burden. This, they argued, would assist the development of appropriate guidelines to manage the disease.

In this context, the current classification of ME/CFS in the *International Statistical Classification of Diseases and Related Health Problems* (ICD), the ICD-10 (10th revision) and ICD-10-CM (Clinical Modification) is confusing and may contribute to this lack of clarity, since the code G93.3, within ‘Diseases of the Nervous System’, specifies ‘post-viral fatigue syndrome’ and is applicable to ‘benign myalgic encephalomyelitis’, while ‘chronic fatigue syndrome’ is an applicable term within ‘chronic fatigue, unspecified’ (R53.82), within the symptoms chapter [10]. The situation may be improved by the intended implementation in 2022 of ICD-11, in which it is proposed to list ‘post-viral fatigue syndrome’ in Chapter 08 (Diseases of the nervous system) under the code 8E49 [11]. While this may create a source of confusion by making an assumption about the aetiology of the condition, which cannot always be demonstrated, this is mitigated and clarified by the addition of ‘benign myalgic encephalomyelitis’ and ‘chronic fatigue syndrome’ as inclusion terms, and of ‘fatigue’ as an exclusion.

In relation to the economic burden of ME/CFS, Brenna and Gitto [8] highlighted that the most substantial component is the indirect costs that arise as a result of productivity losses. Indeed, attempts have been made in the last decade to evaluate the societal costs of ME/CFS, especially in terms of occupational outcomes, such as absenteeism, work incapacity, and early retirement. For example, estimates in the UK population suggest an average yearly productivity loss due to discontinuation of employment of £ 22,684 per patient in the period 2006–2010, with a significant gap between women (£ 16,130) and men (£ 44,515) [12]. To these figures should be added other direct non-medical costs, such as informal care provided by relatives, neighbours or friends, as well as intangible costs related to diminished quality of life. The study also highlighted the need to identify common diagnostic criteria and to alert policy makers to the overall economic burden of this disease (i.e., its impact on society as a whole) and other non-health impacts.

This article reports on work undertaken by Working Group 3 of the European Network on ME/CFS (EUROMENE) in the development of a consistent Europe-wide approach to investigating the economic impact of ME/CFS. It is structured as follows. It starts with an overview of EUROMENE and Working Group 3, followed by a detailed discussion of the challenges and issues involved in developing a consistent Europe-wide approach to measurement that have been identified by the group. After this, we set out a range of recommendations for addressing each of the challenges, while the final section concludes.

## 2. European Network on ME/CFS (EUROMENE)—A European Cost Action Project

The EUROMENE research network was formally established in 2016 as a collaborative, Europe-wide, consortium aiming to address serious gaps in knowledge of ME/CFS. The network now involves 22 countries, with four working groups focused on: epidemiology; biomarkers and diagnostic criteria; clinical research; and socio-economics. All working groups have the active involvement of researchers from across Europe [13].

The intended long-term impact of the EUROMENE collaboration is that of “preventing ME/CFS, determining suitable treatments or avoiding unnecessary treatment in order to improve patients’ quality of life”. In terms of the rationale for a socio-economic work package, we estimated that the annual burden of ME/CFS in Europe, on the basis of extrapolation from UK estimates [14], could be in the region of € 40 bn if the prevalence and cost burden associated with each case were similar to those found in the UK. Therefore, even a modest 1% reduction in the overall burden could deliver cost savings of around €400 million/year, while there may be scope for significantly reducing the economic cost of ME/CFS through effective prevention and treatment, though the costs of such initiatives also need to be recognised. The EUROMENE Action aims to “promote further research on ME/CFS with high economic impact” [15].

In this context, the terms of reference for Working Group 3 (Socio-economics) require it to coordinate efforts to determine the societal impact of ME/CFS, to appraise the economic implications from the disease, and to do so by enabling the estimation of the burden of ME/CFS to society and the provision of long-term trend estimates for societal impact [15]. In addition, specific objectives of the working group include: to survey the existing data from European countries pertaining to economic losses attributable to ME/CFS; to develop approaches to calculating the direct and indirect economic burdens due to ME/CFS; and to provide an integrated outcome assessment framework [15].

## 3. Challenges in Developing a Consistent Europe-Wide Approach to Measuring the Economic Impact of ME/CFS

In this section we describe the progress made by EUROMENE Working Group 3 in the development of a Europe-wide approach to investigating the economic impact of ME/CFS. We start by setting out an overview of the challenges faced, followed by more in-depth discussion of the most relevant issues. In particular, we focus on challenges relating to case definition and prevalence rates, case ascertainment, and the determination of costs, as well as Europe-wide comparisons.

### 3.1. Overview of Challenges

As noted above, there are major challenges rendering economic analyses of ME/CFS problematic, and these include the use of different case definitions and the unwillingness of many doctors to diagnose it. Both of these factors have major impacts on incidence and prevalence estimates, while, in addition, we have established that there is a lack of routinely collected data which could contribute to such estimates. In particular, since a high proportion of patients with ME/CFS remain undiagnosed, there is no obvious way to estimate the costs incurred by such patients. Finally, issues arising in respect of international comparisons of the economic impact of ME/CFS include the heterogeneous nature of patterns of organisation and delivery of health and social care across Europe, differences in the availability of social support and welfare benefits, the varying levels of economic development and wealth of different European countries, and the problem of comparing economic impacts when different currencies are in use in different countries, and are subject to variations in exchange rates.

Our review of the position regarding the economic impact of ME/CFS has led us to identify a number of challenges that need to be addressed if progress is to be made in this area. These issues are summarised in Table 1 and are addressed in more detail in the subsequent sub-sections.

### 3.2. Case Definition and Prevalence Rates

The major problem in determining the overall economic burden of disease attributable to ME/CFS, i.e., little agreement over case definition, has been considered by a number of authors. For example, Brurberg et al. [16] reviewed the comparability of case definitions and identified papers in which different case definitions have been applied to the same patient populations, making possible direct comparisons of the impact differences in case definition have on apparent prevalence. They listed 20 case definitions developed from 1988 onwards, which tend to define different populations and which therefore impact significantly on the perceived prevalence of the disease, and also levels of severity and hence of need for care within the identified patient population.

The case definition most commonly used for research purposes has been that produced by the US Center for Disease Control in 1994, otherwise known as the Fukuda definition [17]. More recently, the Canadian Consensus Criteria (CCC), which identify a more severely affected group of patients than the Fukuda definition, have been widely accepted [3]. A UK study found that some 50% of those patients who conformed to the Fukuda definition conformed also to the CCC [18], while a parallel study in the UK concluded that there were advantages to using both definitions, in order to take advantage of the greater sensitivity of the Fukuda definition and the greater specificity of the CCC [5]. A new case definition has been proposed by the Institute of Medicine (IOM, 2015) [19] that received international recognition. Because of their relative simplicity, the IOM criteria seem useful for screening patients in clinical practice, the CCC criteria being used to confirm the diagnosis of ME/CFS. Working Group 1 of EUROMENE (Epidemiology) proposes that the Fukuda and CCC definitions should be used in all participating European countries [20]. Working Group 3 (Socio-economics) accepts this guidance and also recommends use of a symptom checklist, enabling data to be collected of such a nature that mapping algorithms can be applied to them, enabling conformity to both Fukuda and CCC to be determined. Such a symptom checklist was developed by Osoba et al. [21].

A study in three English regions [18] found a prevalence rate of 0.19% conforming to the CDC-1994 definition in the age group 18–64, but only 0.10% conforming to the more recent Canadian definition [3]. The comparative assessment previously reported by Jason et al. in 2004 [22] found that the Canadian criteria selected cases with less psychiatric co-morbidity, more physical functioning impairment, and more fatigue/weakness, neuropsychiatric and neurological symptoms than the CDC-1994 definition. Two papers comparing the CDC-1994 and Australian definitions are cited. Lindal et al., in Iceland, found population prevalences of 2.1% (CDC-1994) and 7.6% (Australian) respectively [23], while Wessely et al., in England, found that use of the CDC-1994 definition produced a population prevalence of 2.6%, while the equivalent figure using the Australian definition was 1.4% [24]. Brurberg et al. [16] attributed this variation in prevalence obtained using the Australian definition to differences in data collection methods; the CDC-1994 definition appeared more robust and less likely to be affected by variations in data collection methods.

In conclusion, it is clear that, if comparable data on the economic impact of ME/CFS are to be collected across Europe, there needs to be comparable data on the prevalence of the illness, and this in turn requires agreement on case definition. Most of the work done to date in this area has used the CDC-1994 definition [17], which cannot be ignored because of its widespread use in the past but is not ideal for epidemiology. This is because it was not designed for that purpose, but rather to enable well-characterised and relatively homogeneous groups of patients to be identified for clinical trials. As regards cost of illness, one can hypothesise that overall costs, at a national level, will appear greater using the more sensitive CDC-1994 definition, while costs per case will be greater using the Canadian definition [3]. In adopting this two-fold approach, we are acting in concert with the epidemiology and the diagnostic methods/biomarkers working groups of EUROMENE.

### 3.3. Case Ascertainment

As stated above, it is well known that many doctors do not diagnose ME/CFS, further complicating the estimation of accurate prevalence data. It also makes it much more difficult to determine the economic impact associated with the illness when using service utilisation data.

Among the countries participating in the EUROMENE network, the only published work on case ascertainment we have been able to identify comes from Ireland, Belgium, Norway and the UK. In Ireland, Fitzgibbon et al. found that 58% of GPs accepted CFS as a distinct entity in 1997 [25], while in Belgium, a survey of patients attending a fatigue clinic concluded that only 35% of GPs had experience of CFS, with only 23% having sufficient knowledge to treat the condition [26]. A Norwegian study found that the quality of primary care was rated poor by 60.6% of ME/CFS patients [27]. In a survey of 811 GPs in South-West England, with a response rate of 77%, 48% did not feel confident with making a diagnosis of ME/CFS and 41% did not feel confident in treatment, though 72% of GPs accepted ME/CFS as a recognisable clinical entity [28]. Bayliss et al. also found that many GPs lacked confidence and knowledge in diagnosing and managing people with ME/CFS [29]. They made available to GPs an online training module and an information pack for patients, but nearly half of all patients in their study (47%) failed to receive it. Finally, a study in South Wales concluded that the level of specialist knowledge of CFS in primary care was low, and only half the GP respondents in their survey believed that the condition actually existed [30].

As part of our work, we carried out a survey among EUROMENE participants regarding GP diagnosis of ME/CFS. Responses were received from Bulgaria, France, Germany, Ireland, Italy, Latvia, the Netherlands, Norway, Romania, Spain and the UK, and discussed at meetings of the Working Group. Only in Latvia, Norway and the UK was it reported that GPs have lists of all registered patients, which could provide denominator data for primary care-based prevalence studies. In many countries, the proportion of people with ME/CFS presenting to a GP was not known. Where estimates were made, these varied from 20% to 100% per annum. In turn, the proportion of those people with ME/CFS who, having consulted a GP, are referred to specialist care, was estimated at about 60% in Latvia and 80% in Spain. In France, it was thought that the majority were referred, while in the UK it likely varied according to region. The proportion of patients with ME/CFS who self-refer to specialist services was thought to be around 30–40% in Latvia and 80% in Spain. In the UK the figure was thought to be very low, and in most countries this was not known.

Specialist care is highly variable in nature, and different clinical specialties are involved in the different secondary care centres that offer services. In many countries, such services are non-existent. There is official guidance on treatment pathways for ME/CFS in Spain, Norway, the Netherlands and the UK. In Italy and Latvia, the majority of GPs do not recognize ME/CFS as a genuine entity. This is also true of Spain as a whole, though not of Catalonia. In France, it is generally regarded as psychological in nature. In both the UK and the Netherlands it is officially recognised, though many GPs still refuse to accept this. In Catalonia, GPs were said to be confident in diagnosing ME/CFS, but in Latvia, Norway, the Netherlands, France and the UK, there was considerable lack of confidence. The fact that this is a diagnosis made essentially through exclusion of other possible diagnoses undoubtedly contributes to this. The proportion of patients with ME/CFS who consult their GPs and are in fact diagnosed by them was generally said to be low or unknown. In those countries where a proportion was estimated (Spain, France, UK), it was thought to be around 20–50%.

Overall, it is clear that, in Europe, a high proportion of GPs, which is likely to be at least 50%, do not recognise ME/CFS as a genuine clinical entity and therefore do not diagnose it. Among those GPs who do recognise its existence, there is a marked lack of confidence in making the diagnosis and managing the condition. Therefore, estimates of the public health burden of the illness, even where these exist, are likely to underestimate substantially its true prevalence.

### 3.4. Determination of Costs

The overall economic burden of ME/CFS within participating European countries could be determined by the implementation of cost of illness studies. These would have to be prevalence rather than incidence based, since little is known about the prognosis of the disease. There have been Europe-wide cost of illness studies in other conditions, such as cancer [31,32], and the output from such studies can be invaluable, both in informing health and social care policy, and facilitating the management of health and care services. For example, Tarricone states: “COI is a descriptive study that can provide information to support the political process as well as the management functions at different levels of the healthcare organisations. To do that, the design of the study must be innovative, capable of measuring the true cost to society; to estimate the main cost components and their incidence over total costs; to envisage the different subjects who bear the costs; to identify the actual clinical management of illness; and to explain cost variability. In order to reach these goals, COI need to be designed as observational bottom-up studies” [33].

There have been relatively few cost of illness studies of ME/CFS. Those that exist were undertaken in the USA, Australia and the UK, the latter being the only European country where such studies have been carried out. Hunter et al. [34] compared three such studies, by Collin et al. [12], McCrone et al. [14], and Sabes-Figuera et al. [35], and two trials which contained cost data, by McCrone et al. [36], and Richardson et al. [37]. One potential problem in comparing the outcomes of such studies arises from the multiplicity of case definitions that exist for ME/CFS, as noted earlier. Whereas the cost of illness studies by both Collin et al. [12] and McCrone et al. [14] used the Fukuda definition [17], Sabes-Figuera et al. [35] undertook a primary care based study of chronic fatigue (not ME/CFS) and used a case definition not dissimilar to, but less stringent than, that of the National Institute for Health and Clare Excellence (NICE) [38], which is less restrictive than the Fukuda definition [39].

Other studies which did not meet the inclusion criteria for the 2020 Health report [37] include three American studies, by Jason et al. [40], Lin et al. [41] and Reynolds et al. [42]. The study by Jason et al. was an archive-based database study which used the CDC-1994 definition [43], as did the population-based telephone survey by Lin et al. [41]. The study by Reynolds et al. involved analysis of data from a population-based epidemiological study in Wichita, Kansas [43], which also used the CDC-1994 (Fukuda) definition. The final cost of illness study identified was an Australian population-based study by Lloyd and Pender [44], which predated the CDC-1994 definition and used the 1990 Australian definition [6].

A further cost of illness study undertaken in the UK by researchers at Sheffield Hallam University in 2007 for the charity Action for ME surveyed nearly 3000 people with ME/CFS, recruited through patient organisations. It concluded that, at that time, the total costs of ME/CFS could have been over £ 10,000 p.a. per patient, or £ 0.6 billion and £ 2.1 billion per year nationally, depending on the prevalence estimate used [45]. More than 90% of this was due to loss of income, with NHS healthcare costs quite small in comparison, though it was not made clear how cases were defined in the study.

As part of EUROMENE Working Group 3’s activities, a study was undertaken in Latvia to explore to what extent the economic burden of ME/CFS could be determined from routinely collected process data. In order to do this, authors DA and UB made use of data from the Latvian Centre for Disease Prevention and Control (CDPC) and The National Health Service (NHS) of Latvia. Patient-related data were classified by ICD-10 code. ICD-10 codes of interest for this study were G93.3 Post-viral fatigue syndrome, R53 Malaise and fatigue, in particular R53.82 Chronic fatigue, unspecified (which is not identified separately in official statistics), and B94.8 (Sequelae of other specified infectious and parasitic diseases). CDCP data from primary care indicated that approximately 700 patients had ICD-10 code G93.3 assigned, while there were approximately 15,000 with ICD-10 code R53, and about 70 with code B94.8. In toto, these constitute about 0.8% of the Latvian population, which is considerably higher than the prevalence found in other comparable populations. Therefore, it is likely, though unconfirmed, that the category R53 includes a great many patients with illnesses other than ME/CFS. Category G93.3, by contrast, looks like a significant underestimate of the true population prevalence. The total of recorded health system costs for all these categories in 2017 was € 63,893,580, but the authors noted that a great deal of additional data would be required in order to make an accurate determination of the real costs to Latvian society of ME/CFS. These included numbers of confirmed ME/CFS diagnoses, costs of illness and out of pocket treatment costs per patient, patient reported outcomes, the benefits of management of ME/CFS, and return on investment [46].

Overall, on the basis of the activities of Working Group 3 on the determination of costs, we believe any attempt to measure societal economic losses attributable to ME/CFS must take into account direct and indirect costs incurred both by healthcare systems, patients and families, and this applies equally to patients who have been diagnosed as having ME/CFS and those who have not received a diagnosis. As noted, it is likely to be difficult to identify this latter group, for obvious reasons. Furthermore, we also believe that due to variation within the ME/CFS population, it can be hypothesised that, for example, severely affected people (housebound or bedbound) may incur greater costs than mildly or moderately affected people. There is no information available which could shed light on this, which clearly requires further research.

### 3.5. Europe-Wide Comparisons

A comprehensive review of the financing and organisation of health care in the European Union, conducted by WHO for the European Observatory on Health Systems and Policies in 2009, documented in detail the diversity of such arrangements throughout Europe [47]. Similar diversity is found in terms of health outcomes and general levels of health, but no correlation was found between accessibility of health care and funding levels in a somewhat crude analysis [48].

Against such a background, it is clearly a challenge to reach meaningful conclusions about differences in costs and losses attributable to ME/CFS across Europe, particularly given the variety of systems of healthcare delivery that exist, as well as varying stages of economic development. This is because there is a problem making valid comparisons of health care costs between countries which differ markedly in terms of wealth and levels of economic development. We propose that, for ME/CFS, purchasing power parity (PPP) adjustments should be used in any comparisons. This is a method for comparing the price of goods between countries. Using a ‘basket of goods’ of items commonly purchased by consumers, such as bread, milk, and shampoo, PPP is a ratio of the total cost of these goods between two countries. In this way, one can compare what 1 unit of currency can buy across different countries and convert the values back to a single reference currency [49]. For Europe, the obvious choice for the reference currency is the Euro.

In terms of specific data items required for conducting comparable cost of illness studies across countries, the need for more comprehensive data collection at the level of the individual patient is supported by other sources. Jo [50] has itemised the range and scope of the data required to support cost of illness studies, and there may be variation across countries in the availability of data items due to differences in the organisation and funding of health care. These include the data required to identify both system costs and costs to the individual with ME/CFS and those close to him or her. A recent study undertaken in Italy to determine costs to the individual assessed the direct and indirect costs of ME/CFS via a questionnaire distributed via Italian patient associations [51]. By estimating the cost of medical procedures and the cost of lost working time, the study arrived at an estimate for the total economic burden of the disease. The questionnaire was discussed in detail by Working Group 3 both in terms of its specificity and applicability to different countries. This study could, the Working Group felt, be repeated in other countries, in order to enable the acquisition of data capable of direct comparison between countries. The study also aimed to relate the cost impact on people with ME/CFS to their clinical condition and the severity of the disease through the use of the EuroQol-5D instrument to assess health status [52,53]. An alternative approach to achieving this objective could involve the use of instruments for resource use measurement [54,55].

## 4. Recommendations

Our review has led us to a number of conclusions and recommendations as to how research in this area could be advanced. For example, there are a number of serious omissions in the availability of data necessary for investigation of the economic impact of ME/CFS and these are summarised in Table 2. The main areas of concern relate to: case definition; case identification; prevalence and incidence rates; economic cost estimates; data items; and data audits.

In addition, a major problem we have identified is the lack of any information on the economic impact of severe ME/CFS. For this reason, these patients were necessarily excluded from this review, but, since it can be hypothesised that the economic impact of such illness is likely to be greater than that of mild or moderate disease, this is clearly a subject that requires further investigation. It is of considerable importance that this be confirmed empirically, and the scale of any such variation be determined, as a necessary prerequisite to the overall determination of the economic impact of ME/CFS in Europe.

## 5. Conclusions

Problems impeding research into the economic impacts of ME/CFS in Europe include those of arbitrary case definitions, incomplete and inadequate information on incidence and prevalence, failure to diagnose the condition on the part of a high proportion of doctors, and how to determine costs and losses, given variation within the ME/CFS population (e.g., between severely affected and mildly or moderately affected patients, with different cost implications for each of these categories). There are also important issues relating to the heterogeneity of national economies and health care systems within Europe. As a result, we have made a set of recommendations concerning how further progress might be made in this area, in particular in relation to case definition, case identification, descriptive epidemiology, methods of economic investigation, data items required to support such investigation, data audit of quality and completeness, and international comparisons and the compilation of Europe-wide statistics. The relationship between disease severity and economic impact should be a high priority for future research.

## Figures and Tables

**Table 1:** Challenges in assessing the economic impact of ME/CFS in Europe.

1.	Case definition	ME/CFS is a syndrome, defined in terms of its symptomatology rather than its underlying pathology. Work done in this area is therefore dependent on case definitions, which of their very nature are arbitrary. In addition, there are numerous case definitions in use, which vary substantially in sensitivity and specificity, and do not necessarily identify the same population.
2.	Incidence and prevalence	Little is known about the incidence or prevalence of ME/CFS. Very little work has been done in this area in Europe, except in the UK. Conclusions drawn from UK experience, or indeed from work done in other countries, in particular the USA and Australia, cannot be readily extrapolated to Europe as a whole, because the extent of natural variation between populations is unknown.
3.	Failure to diagnose	A high proportion of doctors, in particular GPs, refuse to recognise ME/CFS as a genuine clinical entity, and as a result do not diagnose it. Even in countries where ME/CFS is officially recognised, this proportion may be as high as 50%. It is not possible therefore to obtain accurate prevalence data through the use of service utilisation data.
4.	Determination of costs and losses	Any attempt to determine costs and economic losses attributable to ME/CFS must take into account direct and indirect costs incurred both by healthcare systems, patients and families, as well as productivity losses. This applies equally to patients who have been diagnosed as having ME/CFS and those who have not received a diagnosis, including for the reasons outlined in (iii) above. It is likely to be difficult to identify costs for this latter group, for obvious reasons.
5.	Variation within the ME/CFS population	It can be hypothesised that, for example, severely affected people (housebound or bedbound) may incur greater overall costs than mildly or moderately affected people. There is no information available which could shed light on this, which clearly requires further research.
6.	Heterogeneity of national economies and health care systems	Against such a background, it is clearly an uphill struggle to reach meaningful conclusions about the costs and losses attributable to ME/CFS across Europe, particularly given the variety of systems of healthcare delivery in Europe, and varying stages of economic development.

**Table 2:** Summary of Recommendations.

	Area of Concern	Recommendation
1	Case definition	That there should be Europe-wide adoption of the Fukuda (CDC-1994) case definition alongside the Canadian Consensus Criteria (CCC).
2	Case identification	That a common symptom checklist should be used, capable of being mapped by algorithms onto both the Fukuda case definition and the CCC.
3	Prevalence and incidence	Better descriptive epidemiological information is required, as a basis for economic investigation. This should include information concerning the proportion of severely affected people, as there are likely to be different cost implications for such people, in comparison with those with mild or moderate illnesses.
4	Economic investigation	Prevalence based cost of illness studies, based on these case definitions, should be carried out in different countries, to determine the overall cost burden attributable to ME/CFS.
5	Data items	A list of data items required for cost of illness studies has been identified (though not reported here). Individual participating countries should examine this, to ensure that, insofar as these are derivable from routine data collection, systems are in place to ensure that they are collected.
6	Data audit	The availability in participating countries of the relevant data items referred to above which are required for cost-of illness studies should be examined, with a view to achieving convergence, and facilitating international comparisons.
7	Relationship between disease severity and economic impact	The EuroQol-5D instrument [52,53] should be used as a generic measure of health status and as a multi-attribute utility instrument to determine the relationship, if any, between disease severity and economic impacts, as in the Italian study reported in this document [51], and to inform future economic evaluations in ME/CFS. We further recommend that the Italian study be replicated in other countries, to enable international comparisons to be made.
8	International comparisons and compilation of Europe-wide statistics	Given the diversity of patterns of health care organisations and health funding, as well as of outcomes and general levels of health, and of national wealth and levels of economic development, we recommend the use of purchasing power parities (PPP) in order both to make valid international comparisons and to collate meaningful statistics at a European level.

## Abbreviations

ME/CFS	Myalgic Encephalomyelitys/Chronic Fatigue Syndrome
EUROMENE	European Network on ME/CFS
COI	Cost of illness
EuroQol-5D	An instrument for measuring quality of life
Bn	Billion
CCC	Canadian Consensus Criteria
PPP	Purchasing Power Parity
ICD-10 CM	The international diagnostic classification standard for reporting diseases, disorders, injuries and health conditions for all clinical and research purposes
